# Measuring mammographic density: comparing a fully automated volumetric assessment versus European radiologists’ qualitative classification

**DOI:** 10.1007/s00330-016-4309-3

**Published:** 2016-03-24

**Authors:** Hanna Sartor, Kristina Lång, Aldana Rosso, Signe Borgquist, Sophia Zackrisson, Pontus Timberg

**Affiliations:** 1 Medical Radiology, Department of Translational Medicine, Lund University, Lund, Sweden; 2 Department of Medical Imaging and Physiology, Skåne University Hospital, Inga Marie Nilssons gata 49, SE-205 02 Malmö, Sweden; 3 Epidemiology and Register Centre South (ERC Syd), Skåne University Hospital, Klinkgatan 22, SE-221 85 Lund, Sweden; 4 Division of Oncology and Pathology, Department of Clinical Sciences Lund, Lund University, Lund, Sweden; 5 Department of Oncology, Skåne University Hospital, Getingevägen 4, SE-221 85 Lund, Sweden; 6 Department of Medical Radiation Physics, Department of Translational Medicine, Lund University, Lund, Sweden

**Keywords:** Mammography, Screening, Diagnostic imaging, Breast cancer, Women’ health

## Abstract

**Objectives:**

Breast Imaging-Reporting and Data System (BI-RADS) mammographic density categories are associated with considerable interobserver variability. Automated methods of measuring volumetric breast density may reduce variability and be valuable in risk and mammographic screening stratification. Our objective was to assess agreement of mammographic density by a volumetric method with the radiologists’ classification.

**Methods:**

Eight thousand seven hundred and eighty-two examinations from the Malmö Breast Tomosynthesis Screening Trial were classified according to BI-RADS, 4th Edition. Volumetric breast density was assessed using automated software for 8433 examinations. Agreement between volumetric breast density and BI-RADS was descriptively analyzed. Agreement between radiologists and between categorical volumetric density and BI-RADS was calculated, rendering kappa values.

**Results:**

The observed agreement between BI-RADS scores of different radiologists was 80.9 % [kappa 0.77 (0.76–0.79)]. A spread of volumetric breast density for each BI-RADS category was seen. The observed agreement between categorical volumetric density and BI-RADS scores was 57.1 % [kappa 0.55 (0.53-0.56)].

**Conclusions:**

There was moderate agreement between volumetric density and BI-RADS scores from European radiologists indicating that radiologists evaluate mammographic density differently than software. The automated method may be a robust and valuable tool; however, differences in interpretation between radiologists and software require further investigation.

***Key Points*:**

• *Agreement between qualitative and software density measurements has not been frequently studied.*

• *There was substantial agreement between different radiologists´ qualitative density assessments.*

• *There was moderate agreement between software and radiologists’ density assessments.*

• *Differences in interpretation between software and radiologists require further investigation.*

## Introduction

High mammographic density has consistently been shown to be associated with an increased risk of breast cancer [1]. Hence, there has been a growing interest of evaluating mammographic density for individualized screening programs [2] and for incorporation in risk prediction models [3]. However, optimal use of mammographic density requires a reliable measuring method. Today, both qualitative and quantitative mammographic density measurement methods are available [4]. The most often used clinical classification of mammographic density is the qualitative Breast Imaging-Reporting and Data System (BI-RADS) [5]. Although afflicted with substantial interobserver variability (kappa 0.43–0.79) [6–12], mammographic density as classified by BI-RADS has consistently been associated with an increased risk of breast cancer [1, 13]. However, the latest BI-RADS 5th Edition aims to capture the risk of masking of tumors by dense breast tissue, more than the risk of developing breast cancer [5]. In order to improve objectivity and reproducibility, quantitative breast density measurements have been developed [4]. The area-based, semi-quantitative measurements, such as Cumulus, are represented by different computer-assisted techniques [4]. However, these techniques are also user-dependent and time-consuming. Both the breast itself and the dense breast tissue are three-dimensional, and a previous study reported volumetric breast density measurements to more accurately estimate breast cancer risk than breast density estimated with area-based methods [14]. Previous studies on fully automated volumetric methods of measuring breast density have shown high reproducibility [15] and association with breast cancer risk [16, 17]. Furthermore, the volumetric methods have shown to be positively associated with BI-RADS categories [18–21] as well as to magnetic resonance imaging (MRI) measurements of breast fibroglandular tissue [22, 23]. A previous large study (n = 8867) showed good correlation between two different automated techniques of measuring volumetric breast density, but the agreement with visually estimated mammographic density was poor, albeit better than the agreement with the area-based method [24]. In addition to a mere value or a category of mammographic density, temporal changes in mammographic density have also rendered attention. A decrease of mammographic density has been shown to be associated with a decreased risk of contra-lateral breast cancer [25] as well as a positive marker for response to tamoxifen treatment [26], further motivating a more sensitive measurement than the rather coarse BI-RADS categories.

The aim of this study was to assess the agreement of mammographic density by a fully automated volumetric method with the radiologists’ classification according to BI-RADS 4th Edition. Part of the Malmö Breast Tomosynthesis Screening Trial (MBTST) population, comprising nearly 8500 screening mammography examinations with measured volumetric mammographic density and qualitative classification according to BI-RADS, was used to address the aim of this study.

## Material and methods

### Malmö breast tomosynthesis screening trial (MBTST)

**Fig. 1:**
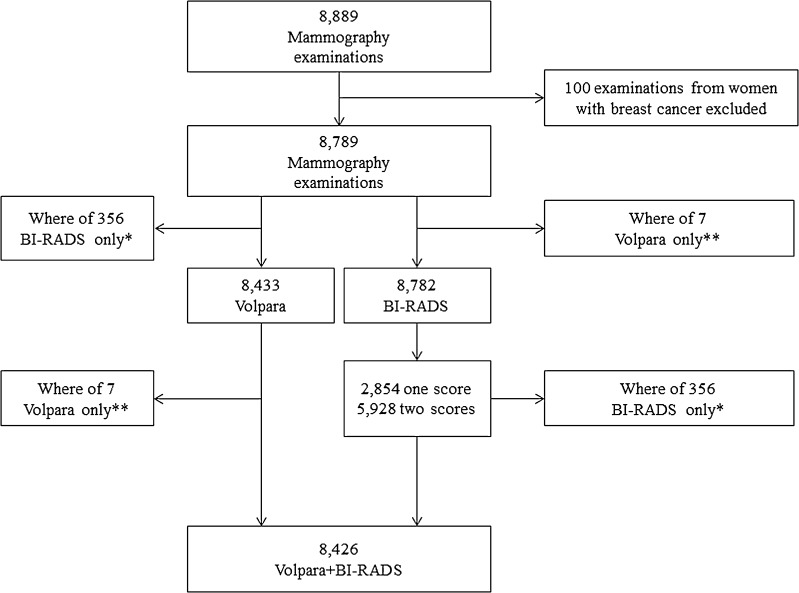
Flowchart illustrating study population. *This represents the same examinations. 1 not included in Volpara file, 22 breast implants, 333 missing Volpara values. **This represents the same examinations. Seven examinations without BI-RADS scores

The MBTST is a prospective study investigating the use of one-view [mediolateral oblique (MLO)] digital breast tomosynthesis (DBT) alone compared to two-view digital mammography [DM; craniocaudal (CC) and MLO] in a population-based screening program in the city of Malmö, Sweden. The MBTST started in January 2010 and results from the first half of the study population have been described in detail previously [27]. Of 10,547 women invited to the first half of the MBTST, 7500 participated in the study, corresponding to a participation rate of 71.1 % [27]. For all DM (Mammomat Inspiration, Siemens AG, Erlangen, Germany), the anode/filter combination was Wolfram/Rhodium and the automatic exposure control was specified to an average glandular dose of 1.2 mGy (for a 53-mm standard breast consisting of 50 % glandular tissue and 50 % fat tissue) [28]. Raw data from the DM examinations were saved on a dedicated server from February 8, 2012 onwards. This present study was based on the DM examinations with available raw data from February 8, 2012 until March 11, 2014. The study population is illustrated in Fig. 1. The examinations from women with breast cancer with at least 10 months of follow up (n = 100) were excluded. Participating women gave written informed consent. This study was approved by the Regional Ethical Review Board at Lund University (Dnr 2009/770) and the local Radiation Safety Board at Skåne University Hospital in Malmö.

### BI-RADS composition categories

A total of 8782 examinations were prospectively classified according to BI-RADS 4th Edition as part of the initial screening reading procedure during the study period. BIRADS 5th Edition was not released at the start of the trial and, hence, it was not used. The following BI-RADS categories for mammographic density were used: BI-RADS 1, almost fat-involuted (<25 % fibroglandular tissue); BI-RADS 2, scattered fibroglandular densities (25–50 % fibroglandular tissue); BI-RADS 3, heterogeneously dense (51–75 % fibroglandular tissue); and BI-RADS 4, extremely dense (>75 % fibroglandular tissue). The scores were performed during the ongoing trial by five breast radiologists, all with more than 10 years of experience in breast radiology. Seven examinations were not evaluated with BI-RADS (Volpara only). A total of 2854 examinations had one BI-RADS score. The study protocol was slightly revised to include double reading of mammographic density; a total of 5928 examinations were independently scored according to BI-RADS by different radiologists resulting in two BI-RADS scores for these examinations.

### Volumetric breast density analyses by Volpara

Volpara measures the x-ray attenuation in relevant parts of the breast and relates it to a region in the breast known to only contain adipose tissue (assuming an even breast thickness). Volpara then produces a fibroglandularity content map of the breast that allows for estimation of breast density measurements. The volumetric breast density refers to the percentage of breast density, computed by dividing the fibroglandular tissue volume by the breast volume. A complete description of the method is found elsewhere [29]. Breast density was measured as a continuous variable [volumetric breast density (VBD); ranging from 0 % to approximately 40 % fibroglandular tissue] as well as four ordered categories [Volpara density grade (VDG)]: VDG 1: < 4.5 % fibroglandular tissue, VDG 2: ≥ 4.5 and < 7.5 % fibroglandular tissue, VDG 3: ≥ 7.5 and < 15.5 % fibroglandular tissue, VDG 4: ≥15.5 % fibroglandular tissue. The thresholds of the VDG categories have been determined by an American expert group of radiologists by recording the average VBD for the assigned BI-RADS category in 500 mammography examinations [29]. The volumetric breast density result was provided per examination by averaging values from the two DM projections (CC and MLO) from both breasts.

A total of 8433 examinations with raw two-view DM data were assessed with a fully automated volumetric breast density measurement software; Volpara (version 1.5.11, Matakina Technology, Wellington, New Zealand). The software calculation was based on four images in 92.4 % of the examinations, but for a few examinations, a lesser amount of images or additional images were assessed (e.g., women with only one breast or software failure for single images). Examinations with previously known breast implants were excluded because the software had known difficulties in correctly measuring volumetric breast density in these images. A total of 356 examinations were not included in analyses with Volpara, predominantly due to lack of DM raw data (Fig. 1). Very few examinations had missing Volpara values due to software failure (≤5 cases)

### Statistical methods

Linear-weighted kappa and 95 % confidence interval (CI) values were calculated for estimation of inter-observer variability for examinations with two BI-RADS scores. Agreement between VBD (continuous variable) and BI-RADS scores was analyzed descriptively. Kappa values for comparison between VDG (categorical variable with four groups) and BI-RADS scores were calculated using a meta-analysis which means that a separate kappa coefficient was calculated for each reader (reader vs. Volpara). The results were then combined by taking the individual kappa estimates into account and weighting them using the standard error for each kappa, rendering a pooled kappa [30]. By convention, values of <0.0, 0.00–0.20, 0.21–0.40, 0.41–0.60, 0.61–0.80 and 0.81–1.00 are, respectively, indicative of poor, slight, fair, moderate, substantial and almost perfect agreement [31]. For examinations with two BI-RADS scores, the score from the first radiologist was used. In an additional sensitivity analysis, the score from the second radiologist instead of the score from the first radiologist was used which did not affect the results. In addition, the radiologists were randomly assigned to be reader one or two. For categorical variables, the percentages of cases in which both methods (or both radiologists) agreed were calculated (i.e., observed agreement). Examinations from women with breast cancer (n = 100) were excluded in all of the analyses. All the calculations were performed using the software Stata v13 (StataCorp LP, Texas, USA).

## Results

### Baseline variables

The mean age at the study mammography examination was 58 years (range 40–76 years). Regarding Volpara density values per examination, the median breast volume was 691.1 cm^3^ (range 40.1–3375.8 cm^3^), the median fibroglandular tissue volume was 49.0 cm^3^ (range 8.8–336.6 cm^3^), and the median VBD was 7.2 % (range 1.9–43.3 %; Table 1). The examinations with VDG classification were distributed as follows: VDG 1: 20.9 %, VDG 2: 32.1 %, VDG 3: 31.5 %, VDG 4: 15.5 %, with a corresponding BI-RADS distribution: BI-RADS 1: 16.4 %, BI-RADS 2: 40.9 %, BI-RADS 3: 35.2 %, BI-RADS 4: 7.5 %.

**Table 1:** Volpara values per BI-RADS category per examination (median, min/max)

BI-RADS^a^ category^b^	n	Fibroglandular tissue volume (cm^3^)	Min/max	Breast tissue volume (cm^3^)	Min/max	Volumetric Breast density (%)	Min/max
1	1378	41.0	12.1/123.2	1005.7	139.1/3188.8	4.1	1.9/26.2
2	3445	44.1	11.5/184.7	777.5	69.6/3375.8	5.7	2.0/32.5
3	2967	60.9	8.8/257.5	567.0	40.1/2831.0	10.9	2.9/32.4
4	636	77.3	13.4/336.6	360.9	56.2/1931.2	22.1	5.1/43.3
Total^c^	8426						
All examinations with Volpara measures^d^	8433	49.0	8.8/336.6	691.1	40.1/3375.8	7.2	1.9/43.3

^a^Breast Imaging Reporting and Data System

^b^BI-RADS score from one reader

^c^Examinations with BI-RADS score from one reader and Volpara values

^d^Independent of BI-RADS scores

### Agreement analyses

**Fig. 2:**
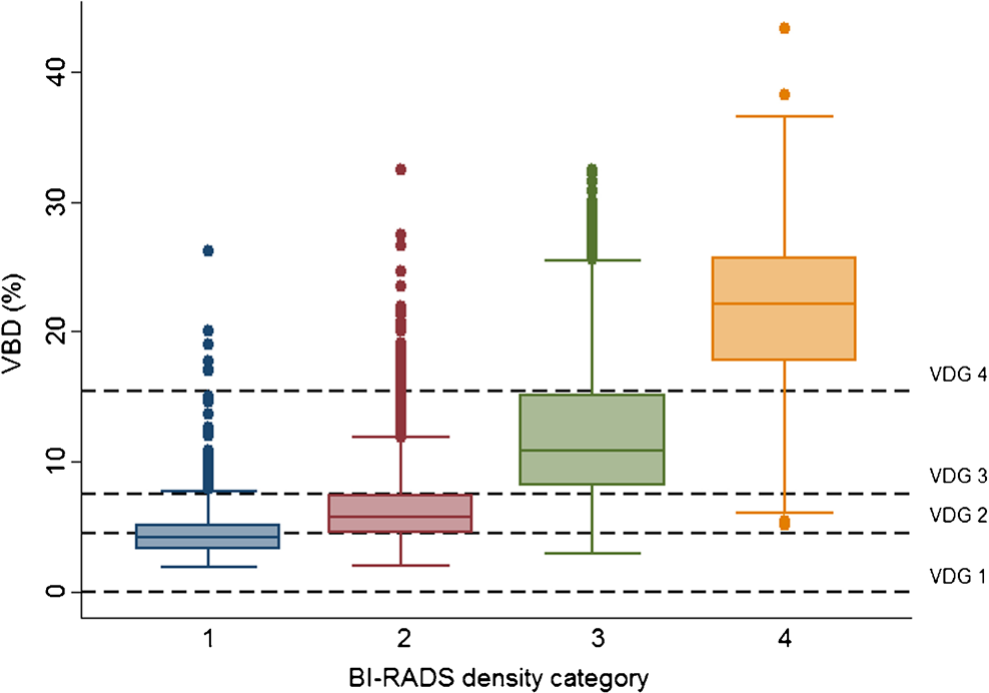
Box plot showing associations between volumetric breast density (VBD) and BI-RADS

**Fig. 3:**
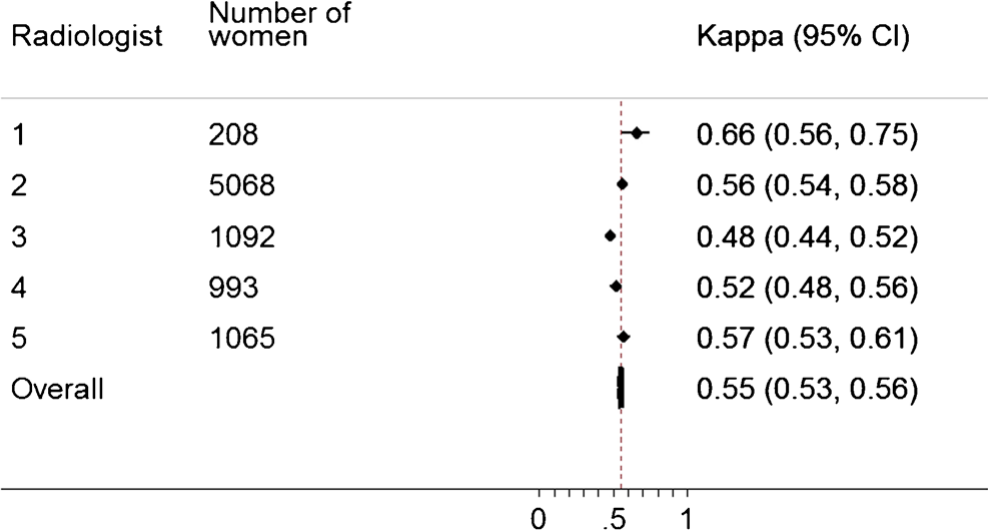
Agreement between Volpara density grade (VDG) and BI-RADS per radiologist

There was substantial agreement between BI-RADS scores with a weighted kappa of 0.77 (0.76–0.79; observed agreement 80.9 %). The distribution of VBD values in relation to BI-RADS categories is shown in Fig. 2. There was a spread of VBD values across each BI-RADS category which might be called poor agreement (Fig. 2). If these two methods of mammographic density measurement were in agreement, we would observe only a certain range of VBD values in each BI-RADS category. There was moderate agreement between VDG and BI-RADS, with a pooled kappa for all five radiologists of 0.55 (0.53–0.56; observed agreement 57.1 %; Fig. 3). Information regarding which of the categories exhibited the most agreement is shown in Table 2; agreement was highest in BI-RADS 4 and similar for the other groups (BI-RADS 1: 60.9 %, BI-RADS 2: 50.2 %, BI-RADS 3: 57.3 %, BI-RADS 4: 85.1 %). Figures and tables show the crude distribution of human labelling errors without corrections. For a few examinations in the data set (n = 6), the BI-RADS scores and VDG values were discrepant over three categories (BI-RADS 1 vs. VDG 4). When specifically looking into those examinations, the BI-RADS scores were believed to be labelling errors by the radiologists.

**Table 2:** Cross tabulation between BI-RADS scores and Volpara density grade (VDG)

	BI-RADS^a^				
VDG^b^	1	2	3	4	Total
1	839	872	52	0	1763
2	457	1730	513	8	2708
3	76	788	1699	87	2650
4	6	55	703	541	1305
Total	1378	3445	2967	636	8426

^a^Breast Imaging Reporting and Data System

^b^Volpara Density Grade

## Discussion

In this large study, we analyzed mammographic density assessment in a screening population with a fully automated volumetric assessment using Volpara software compared to the radiologists’ classification according to BI-RADS, 4th Edition. We found that the agreement between BI-RADS scores was substantial, indicating that the radiologists evaluated the mammographic density in a similar manner. Agreement between VDG and BI-RADS scores was moderate.

Our results are in line with a previous large study showing that different mammographic density measurements did not produce identical results [24]. Morrish et al. showed a low correlation between Volpara and observers’ visual estimations of mammographic density using the VAS method (Visual Analog Scale), albeit better with volumetric density than with area density [24]. Other studies have shown positive associations [21] and good correlations between VBD and BI-RADS [18, 19]. However, the use of correlation instead of agreement in previous studies makes direct comparison with this present study difficult. Furthermore, correlation may not be the method of choice since correlation only measures the strength of a relation between two variables, not the agreement between them [32, 33]. However, there was a difference in mammographic density distribution according to BI-RADS between previous studies [18, 19] and this present study, which may be caused by differences in both age and ethnicity. Asian ethnicity and younger age are known to be associated with higher mammographic density [13, 34] as could be observed in the aforementioned studies. Gweon et al. reported 62 % of the examinations to be categorized as BI-RADS 3 and 18.8 % to be categorized as BI-RADS 4 in an Asian population with a mean age of 51.7 years [18]; the corresponding distribution for this study would be 35.2 % for BI-RADS 3 and only 7.5 % for BI-RADS 4 and a mean age of 58 years. The observations of this study, that Volpara classified more examinations in the highest VDG category than the radiologists (BI-RADS) and that there was moderate agreement between VDG and BI-RADS, have also been previously described [18, 19, 22]. On the other hand, a previous Dutch study reported the BI-RADS distribution to be quite comparable with the VDG distribution, with a weighted kappa value of 0.80 [21].

There could be several explanations for the lower degree of agreement between Volpara and BI-RADS assessments. First, BI-RADS scores are set based on processed images, while Volpara analyses are performed on raw DM data. Second, VBD is measured on a continuous scale and BI-RADS scores are a coarse estimation into four groups. Therefore, values of mammographic density near the limits in the different VDG categories could be classified into the upper or the lower adjacent BI-RADS category since small differences in mammographic density would not be detected by the radiologists. And finally, both Volpara and the radiologists estimate the amount or percentage of dense breast tissue. However, despite the BI-RADS 4th Edition definitions, it might be that the radiologists are also taking into account the distribution of the mammographic density and the difficulty of detecting a breast tumour, which may not always represent an actual increased amount of dense tissue, albeit a previous study reported high volumetric density to be correlated to decreased mammography sensitivity [35]. Taken together, this may indicate that radiologists evaluate mammographic density differently than automated software.

The automated method may still be a robust and valuable tool. High mammographic density, whether measured by Volpara or qualitatively with BI-RADS, has been shown to be associated with an increased breast cancer risk [1, 16, 17]. Previous reviews on mammographic density [2] and breast cancer risk prediction [3] have emphasized the need for improved and individualized breast cancer screening programs and risk prediction models. One way of improving these programs and models could be by incorporating a fully automated volumetric assessment of continuously measured mammographic density that may reduce the interobserver variability [15] and thereby producing a more reliable density estimate. A more reliable density estimate may then be used to stratify women in to different screening and risk groups.

Some limitations of this study require consideration. First, the BI-RADS 4th Edition was standard according to the time period during the main part of the MBTST; the impact of the BI-RADS 5th Edition on the results would have been interesting to analyze. This was, however, out of scope for this study. Second, two previous studies investigating BI-RADS agreement had several radiologists reading the images in the density analyses, which, of course, would have been preferable (11 [11] and 21 radiologists [12]). However, five radiologists is still a realistic number of readers in a single-centre study. Third, breast tumours are known to possibly affect the surrounding breast tissue and, thereby, perhaps also the mammographic density and we, therefore, excluded examinations from women with breast cancer. Finally, consistently registered information on previous breast surgery, use of hormone replacement therapy, or reproductive information was not available, all of which are factors known to possibly affect the mammographic density. However, we do not believe this affected our results because the aforementioned factors are not expected to affect the modes of assessment differently.

The population in this study was a screening population representative of the female population in the screening ages 40–74 years in the city of Malmö, Sweden [27]. Furthermore, the BI-RADS scores were prospectively performed by several radiologists, representing the common mass screening setting. The interobserver variability was low, reflecting a solid evaluation of qualitatively estimated mammographic density. Altogether, this study may well represent everyday screening practice.

In conclusion, there was moderate agreement between Volpara and BI-RADS scores from European radiologists, indicating that radiologists evaluate mammographic density differently than automated software. However, the automated method may still be a robust and valuable tool. In addition to this, the differences in interpretation between radiologists and software will require further investigation. Future studies evaluating fully automated density assessments on different populations are warranted in order to ensure accurate reflection of mammographic density, with an additional focus on breast cancer risk and screening outcomes.

## Abbreviations

BI-RADS: Breast Imaging-Reporting and Data System
CC: Craniocaudal
DM: Digital mammography
DBT: Digital breast tomosynthesis
MBTST: Malmö Breast Tomosynthesis Screening Trial
MLO: Mediolateral oblique
